# Antimicrobial Resistance, Genetic Lineages, and Biofilm Formation in *Pseudomonas aeruginosa* Isolated from Human Infections: An Emerging One Health Concern

**DOI:** 10.3390/antibiotics12081248

**Published:** 2023-07-29

**Authors:** Adriana Silva, Vanessa Silva, María López, Beatriz Rojo-Bezares, José António Carvalho, Ana Paula Castro, Yolanda Sáenz, Gilberto Igrejas, Patrícia Poeta

**Affiliations:** 1 MicroART-Microbiology and Antibiotic Resistance Team, Department of Veterinary Sciences, University of Trás-os-Montes and Alto Douro (UTAD), 5000-801 Vila Real, Portugalvanessasilva@utad.pt (V.S.); 2 Department of Genetics and Biotechnology, University of Trás-os-Montes and Alto Douro (UTAD), 5000-801 Vila Real, Portugal; 3 Functional Genomics and Proteomics Unit, University of Trás-os-Montes and Alto Douro (UTAD), 5000-801 Vila Real, Portugal; 4 Associated Laboratory for Green Chemistry (LAQV-REQUIMTE), University NOVA of Lisboa, 1099-085 Lisboa, Portugal; 5 Área de Microbiología Molecular, Centro de Investigación Biomédica de La Rioja (CIBIR), 26006 Logroño, Spain; 6 Medical Center of Trás-os-Montes e Alto Douro E.P.E., 5000-508 Vila Real, Portugal; 7 Veterinary and Animal Research Centre (CECAV), University of Trás-os-Montes and Alto Douro (UTAD), 5000-801 Vila Real, Portugal; 8 Associate Laboratory for Animal and Veterinary Sciences (AL4AnimalS), 5000-801 Vila Real, Portugal

**Keywords:** *Pseudomonas aeruginosa*, multidrug resistance, resistance genes, virulence genes, biofilm formation, genetic lineages

## Abstract

*Pseudomonas aeruginosa* (*PA*) is a leading nosocomial pathogen and has great versatility due to a complex interplay between antimicrobial resistance and virulence factors. *PA* has also turned into one the most relevant model organisms for the study of biofilm-associated infections. The objective of the study focused on analyzing the antimicrobial susceptibility, resistance genes, virulence factors, and biofilm formation ability of thirty-two isolates of *PA. PA* isolates were characterized by the following analyses: susceptibility to 12 antimicrobial agents, the presence of resistance genes and virulence factors in PCR assays, and the quantification of biofilm production as evaluated by two distinct assays. Selected *PA* isolates were analyzed through multilocus sequence typing (MLST). Thirty *PA* isolates have a multi-resistant phenotype, and most of the isolates showed high levels of resistance to the tested antibiotics. Carbapenems showed the highest prevalence of resistance. Various virulence factors were detected and, for the quantification of biofilm production, the effectiveness of different methods was assessed. The microtiter plate method showed the highest accuracy and reproducibility for detecting biofilm-producing bacteria. MLST revealed four distinct sequence types (STs) in clinical *PA*, with three of them considered high-risk clones of *PA*, namely ST175, ST235, and ST244. These clones are associated with multidrug resistance and are prevalent in hospitals worldwide. Overall, the study highlights the high prevalence of antibiotic resistance, the presence of carbapenemase genes, the diversity of virulence factors, and the importance of biofilm formation in PA clinical isolates. Understanding these factors is crucial for effective infection control measures and the development of targeted treatment strategies.

## 1. Introduction

The ubiquitous gram-negative bacterium *Pseudomonas aeruginosa* is one of the greatest challenges to be overcome in the adequate treatment of nosocomial infections among immunocompromised patients. It is an opportunistic human pathogen associated with chronic and acute life-threatening infections such as cystic fibrosis, cancer, traumas, sepsis, burns, and ventilator-associated pneumonia, among others [1,2]. Antimicrobial resistance became a problem in dealing with infections caused by *P. aeruginosa* as this pathogen began to demonstrate resistance to a variety of antibiotics classes, thus becoming harder to treat regarding morbidity and mortality [3]. Because of this selective pressure, the number of multidrug-resistant strains and their prevalence are increasing worldwide, with rates between 15 and 30% leading to them being considered as one of the greatest challenges of the 21st century. In 2017, the World Health Organization (WHO, Geneva, Switzerland) published a list of antibiotic-resistant “priority pathogens” according to urgency of necessity in terms of the development of new antibiotics, with carbapenem-resistant *P. aeruginosa* number two in the “critical” category and thus considered a public health priority in terms of the development of new antibiotics and therapeutic approaches [1,4]. Antimicrobial resistance is a prominent global health concern that exemplifies the “One Health approach” as it impacts human, animal, and environmental well-being. To address this issue, various global health organizations and governments have taken steps to combat antimicrobial resistance, introducing the “One Health approach”. This approach necessitated a collaborative effort among different disciplines and specialized agencies, such as the Food and Agriculture Organization of the United Nations (FAO, Rome, Italy) and the World Organization of Animal Health (OIE, Paris, France). By working together and leveraging their respective expertise, these agencies aim to mitigate the potential impacts of antimicrobial resistance [5,6,7].

Carbapenemases constitute a group of enzymes that possess the ability to hydrolyze β-lactams and confer the largest antibiotic-resistant spectrum [8,9,10,11]. The emergence and exponential spread of multidrug-resistant (MDR) *P. aeruginosa* strains made it necessary to apply typing methods that allow for a more complete analysis of the spread of MDR strains and the integration of surveillance programs [12]. Multilocus sequence typing (MLST) is a portable, unambiguous, DNA sequence-based technique widely used for molecular typing of bacteria and has identified international clonal complexes responsible for the dissemination of *P. aeruginosa* worldwide [13,14,15]. MDR clones are widely distributed worldwide and possess remarkable adaptability, allowing them to survive in diverse environments on various surfaces, including soils, plants, water, and medical devices. These clones employ influential binding factors such as flagella, pili, and biofilms which enable their attachment [16]. Notably, chronic infections are often attributed to bacterial biofilm formation rather than the planktonic form of bacteria which typically causes acute infections. The transition from planktonic form to biofilm form in flagellated microorganisms is regulated by the quorum-sensing system. Multiple mechanisms associated with biofilm formation are linked to the expression of established genes encoding virulence factors [2,17]. *P. aeruginosa* demonstrates the ability to withstand the immune system response and antimicrobial agents. In the USA alone, approximately 6 million patients are reported to have *P. aeruginosa*-infected wounds each year, with the global cost of these infections exceeding billions of USD. Treating these infections often requires prolonged interventions in an attempt to eliminate the biofilm or increase its susceptibility to treatment [18]. *P. aeruginosa* possesses virulence factors that enhance its fitness and ability to thrive within a human host. These virulence factors play a crucial role in promoting bacterial growth and survival by manipulating host cellular processes, resulting in severe damage, tissue necrosis, evasion of host defenses, and impairment of the immune system [16]. The genome of *P. aeruginosa* has high complexity and variability which can therefore alter and determine resistance determinations that allow it to subsequently survive and spread in different conditions and ecological niches [12]. According to estimates, ST111, ST175, and ST235 are cited as international high-risk clones and are widely spread STs of *P. aeruginosa* in the clinical environmental [12,14].

The aim of this study was to enhance comprehension of the molecular epidemiology and antimicrobial resistance of *P. aeruginosa* strains obtained from different clinical origins in a northern Portuguese hospital. Additionally, the study aimed to characterize the isolates in terms of antimicrobial resistance genes, virulence factors, biofilm production capacity, and genetic lineages.

## 2. Materials and Methods

### 2.1. Samples and Bacterial Isolates

Samples were collected from 32 patients from a northern Portuguese hospital (Centro Hospitalar de Trás os Montes e Alto Douro, CHTMAD, Vila Real, Portugal) from 2018 to January 2020. *P. aeruginosa* strains were isolated from urine, healing liquids, bronchial secretions, blood, pus, drainage liquids, expectoration, and other liquids. Confirmation of *P. aeruginosa* species was carried out through growth on Pseudomonas agar base plates (Liofilchem, Rosetodegli, Abruzzi, Italy) at 37 °C for 24–48 h and by polymerase chain reaction (PCR) to amplify the 249 base pair (bp) of the oprI gene and the 504 bp of the oprL gene using specific primers as previously described [19].

### 2.2. Antimicrobial Susceptibility Testing and Resistance Genes

The susceptibility of the isolates was tested on Mueller–Hinton agar using the Kirby–Bauer disc diffusion method according to European Committee on Antimicrobial Susceptibility Testing (EUCAST, 2020) standards against the following 12 antimicrobial agents: piperacillin (30 µg), ticarcillin-clavulanic acid (75–10 µg), cefepime (30 µg), ceftazidime (10 µg), doripenem (10 µg), imipenem (10 µg), meropenem (10 µg), aztreonam (30 µg), ciprofloxacin (5 µg), amikacin (30 µg), gentamicin (10 µg), and tobramycin (10 µg). DNA extraction from Pseudomonas strains was performed according to the “Boiling method” and was quantified using the ND-100 Spectrophotometer (NanoDrop^®^ spectrophotometer).

According to the carbapenem resistance profile, each isolate was screened for the presence of *bla*_VIM-2_ and *bla*_NDM_ metallo-beta-lactamase (MBL) genes and the *bla*_KPC_ class A carbapenemases gene. The presence of virulence factor genes, toxA (exotoxin A), algD (alginate), plcH and plcN (phospholipases C), and pilA (pili) was determined in all isolates through PCR using specific primers as previously described [20].

### 2.3. Molecular Typing of Selected P. aeruginosa Isolates

*P. aeruginosa* isolates were typed by MLST that was performed by amplifying and sequencing the amplicons of 7 housekeeping genes (acsA, aroE, guaA, mutL, nuoD, ppsA, and trpE). The protocol described on PubMLST (public databases for molecular typing and microbial genome diversity) was followed, and the allele combination was determined after sequencing of the seven genes to determine the sequence type (ST).

### 2.4. Quantification of Biofilm Formation

The biofilm-producing *P. aeruginosa* strains were detected by two different methods: the Congo red agar (CRA) method and the microtiter plate-based method (MM). These methods were used to assess the biofilm-forming capabilities of the strains. Freeman et al. (1989) described the CRA method as a simple qualitative method to detect and evaluate the biofilm formation of *P. aeruginosa* strains. This method utilizes Congo red dye as a pH indicator, showing black coloration when the strains have the ability to form biofilms [21,22]. The MM is a qualitative assessment of biofilm formation and was performed as previously described with some modifications [23]. Two colonies were transferred from recently grown cultures to tubes containing 3 mL of Tryptic Soy Broth (TSB, Oxoid, Basingstoke, UK) and then placed in an incubator at 37 °C for approximately 16 h with continuous shaking at 120 rpm using an ES-80 Shaker-Incubator (Grant Instruments, Cambridge, UK). Following the incubation period, the bacterial suspension was adjusted to an optical density equivalent to 1 Χ 106 colony-forming units. Subsequently, 200 μL of the bacterial suspension from different isolates was added to each well of a 96-well flat-bottom microplate. A negative control consisting of fresh medium without any bacterial inoculum was included. The microplates were then incubated at 37 °C for 24 h without shaking. Each experiment was performed in triplicate.

The quantification of biofilm mass was performed using the Crystal Violet (CV) staining method following the protocol described by Peeters et al. (2008) [24] with some modifications. After the incubation period, the plates were washed twice with 200 μL of distilled water to eliminate non-adherent bacterial cells and were then allowed to air dry at room temperature for approximately 2 h. Subsequently, 100 μL of methanol (VWR International, Carnaxide, Portugal) was added to each well and incubated for 15 min to fix the microbial biofilm. The methanol was removed and the plates were left to dry for 10 min. The CV solution was the discarded, and the plates were washed twice with distilled water to remove excess dye. Following this, 100 μL of 33% (*v*/*v*) acetic acid was added to each well to solubilize the CV dye, and absorbance was measured at 550 nm using a BioTek ELx808U microplate reader (BioTek, Winooski, VT, USA)[24].

## 3. Results

### 3.1. P. aeruginosa Identification and Antimicrobial Resistance Phenotypes

**Figure 1:**
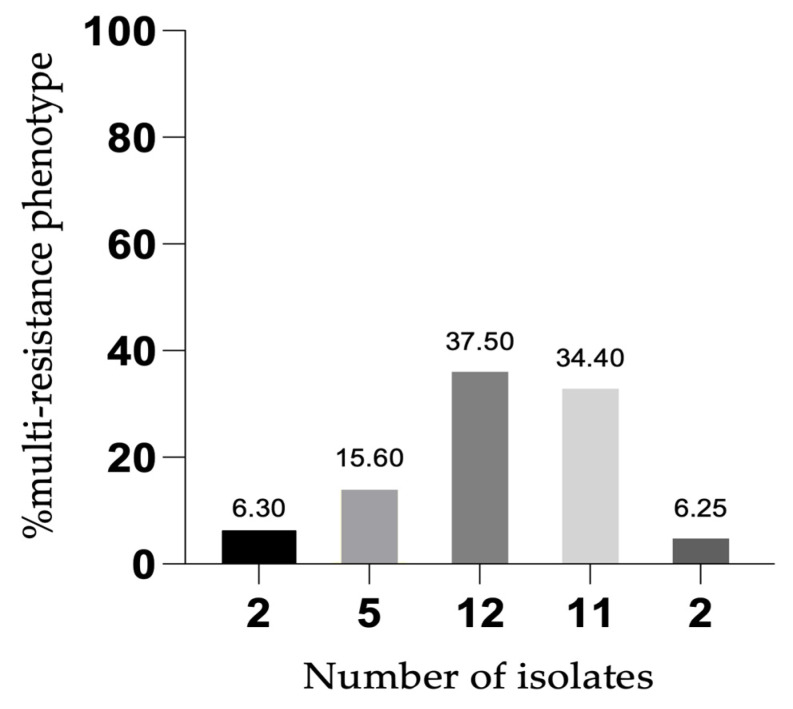
Percentage of multi-resistant phenotypes among the clinical *P. aeruginosa* strains.

Thirty-three clinical samples of Pseudomonas isolates were collected in a local hospital in the north of Portugal from various specimens. *P. aeruginosa* was detected in 32 out of 33 clinical samples. *P. aeruginosa* strains were further characterized for 12 antipseudomonal antibiotics. Thirty (93.8%) of the *P. aeruginosa* isolates studied had a multi-resistant phenotype. Regarding the 32 isolates studied, 2 isolates (6.3%) exhibited resistance to two classes of antibiotics, 5 isolates (15.6%) exhibited resistance to three classes of antibiotics, 12 isolates (37.5%) exhibited resistance to four classes of antibiotics, 11 isolates (34.4%) were resistant to five classes of antibiotics, and 2 isolates (6.25%) showed resistance to six classes of antibiotics (Figure 1).

In the susceptibility testing of clinical isolates, three aminoglycoside antibiotics were tested: tobramycin (TOB), gentamicin (CN), and amikacin (AK). Among the tested isolates, twelve were resistant and twenty were susceptible to TOB, while ten were resistant and twenty-two were susceptible to CN. Only one isolate showed resistance to AK, with thirty-one isolates being susceptible. Additionally, two antibiotic classes were assessed: fluoroquinolones, represented by ciprofloxacin (CIP), and monobactams, represented by aztreonam (ATM). Among the isolates, twenty were resistant and twelve were susceptible to CIP, while twelve were resistant and twenty were susceptible to ATM. Carbapenem susceptibility testing was conducted for three antibiotics: doripenem (DOR), meropenem (MER), and imipenem (IMI). All thirty-two clinical isolates displayed resistance to IMI and a high resistance rate was observed for MER, with twenty-two resistant and ten susceptible isolates. Similarly, twenty-one isolates were resistant and eleven were susceptible to DOR. The cephalosporin class was assessed using two antibiotics: ceftazidime (CAZ) and cefepime (FEP). Ten isolates were resistant and twenty-two were susceptible to CAZ, while twenty-seven were resistant and five were susceptible to FEP. Lastly, the penicillin class was analyzed using two antibiotics: ticarcillin-clavulanic acid (TCC) and piperacillin (PRL). Twenty-one isolates were resistant to PRL, while eleven were susceptible. For TCC, fifteen isolates were resistant and seventeen were susceptible. Many isolates displayed resistance to PRL, TCC, FEP, CIP, DOR, MER, and IMI, which respectively belong to the penicillin, cephalosporin, fluoroquinolone, and carbapenem classes. However, amikacin demonstrated the highest activity among isolates (96.9% susceptibility), followed by gentamicin and ceftazidime (susceptibility rate of 68.6%). Tobramycin and aztreonam exhibited the same susceptibility rate, with 62.5% of isolates being susceptible. Notably, the carbapenem class displayed the highest prevalence of resistance (78.1%) among the clinical isolates examined.

### 3.2. Characterization of Resistance Genes and Virulence Genes

All of the 32 clinical *P. aeruginosa* isolates were resistant to imipenem, and the presence of *bla*_VIM-2_, *bla*_NDM_, and *bla*_KPC_ genes was evaluated through PCR. The *bla*_KPC_ gene was amplified in six isolates (18.8%), and the prevalence of MBL genes was relatively high in the current study, where seven isolates (21.8%) harbored the *bla*_NDM_ gene and three isolates (9.3%) the *bla*_VIM-2_ gene. Regarding the virulence genes, the most detected virulence genes in our 32 *P. aeruginosa* isolates were plcH (78.1%), toxA (65.6%), and algD (43.8%).

### 3.3. Biofilm Formation

The CRA test showed low positivity in the presumptive detection of biofilm production in one isolate with moderate biofilm formation. In the MM, the number of isolates which showed high biofilm formation was three (9.0%), the number showing moderate biofilm formation was thirty (93.7%), and there were no isolates showing no or weak biofilm production. In the current study, the CRA method and MM detected biofilm formation in 6.0% (2/33) and 100% (33/33) of the isolates, respectively (Table 1)**.**

### 3.4. Molecular Typing

The population structure of the 18 *P. aeruginosa* isolates selected, as studied by MLST, showed that the isolates included four sequence types (STs) (Table 2). The most predominant was ST175 (n = 11, 61.11%), followed by ST244 (n = 3, 16.67%), ST2333 (n = 2, 11.11%), and ST235 (n = 2, 11.11%).

## 4. Discussion

*P. aeruginosa* is responsible for infections in various areas, including infections in the urinary tract, bed ulcers, burns, and respiratory infections. Despite advancements in infection control protocols and medical care, *P. aeruginosa* continues to be a significant source of nosocomial infections [25,26]. In our study, *P. aeruginosa* was isolated from patients with urinary tract infections, as well as from healing liquids, bronchial secretions, blood, pus, drainage liquid, expectorations, and other liquids. In the susceptibility testing of clinical isolates, various antibiotics were evaluated against *P. aeruginosa*. The results revealed a significant prevalence of resistance among the tested antibiotics, particularly in the carbapenem class. Resistance was observed in imipenem, meropenem, and doripenem, indicating a challenging situation for the treatment of infections caused by *P. aeruginosa*. On the other hand, aminoglycosides such as tobramycin, gentamicin, and amikacin showed a relatively higher susceptibility rate. Amikacin exhibited the highest activity, followed by gentamicin and ceftazidime. Several studies conducted worldwide have reported a concerning prevalence of antibiotic resistance in *P. aeruginosa*. For instance, a study conducted by Reza Heidari et al. [27] in Iran found high resistance rates of 70.6% for amikacin and 65.7% for ciprofloxacin. Cephalosporins, carbapenems, aminoglycosides, and fluoroquinolones are considered important groups of anti-pseudomonal agents [27]. Another study also described the high prevalence of resistance to antibiotics and MDR strains. In this study carried out by Kiyaga et al. [28], it was revealed that there was a 31% prevalence of MDR strains among the *P. aeruginosa* isolates, and this rise in prevalence reflects the global trend of increasing MDR *P. aeruginosa* infections [29]. Numerous studies provide further evidence of the escalating problem of antibiotic resistance in *P. aeruginosa* infections [30,31,32]. Unfortunately, the antibiotics that were once effective in treating these infections have now become ineffective. These findings suggest that the choice of antibiotic therapy should be carefully considered due to the specific patterns of *P. aeruginosa*.

This study found that all the isolates of *P. aeruginosa* were resistant to imipenem. PCR analysis was conducted to assess the presence of three specific resistance genes: *bla*_VIM-2_, *bla*_NDM_, and *bla*_KPC_. The results demonstrated that 18.8% carried the *bla*_KPC_ gene, and a relatively high prevalence of MBL genes was observed, with 21.8% of the isolates containing the *bla*_NDM_ gene and 9.3% harboring the *bla*_VIM-2_ gene. The KPC (Klebsiella pneumoniae carbapenemase) enzyme is encoded by a gene located on mobile elements such as plasmids, which favor its transference among *P. aeruginosa* and other species. KPC was first reported in Colombia (2007) and later in other countries in the American continent, where recently an increasing frequency of KPC-producing *P. aeruginosa* has been reported in hospitals [33,34,35]. In our study, there was a prevalence of 18.8%; other studies carried out in other continents also identified the presence of these genes in isolates. A study carried out in Brazil detected the presence of this gene in 33.3% of the isolates, and these isolates were characterized as being imipenem- and/or meropenem-resistant isolates [36]. In another study, samples from intensive care unit (ICU) burns patients collected from August 2017 to August 2018 were analyzed, and the presence of *bla*_KPC_ carbapenemases was detected in 27.3% of the analyzed isolates [37]. Recent investigations [38] have also identified the presence of these genes, and these findings emphasize that these genes are involved in the resistance mechanism exhibited by *P. aeruginosa* [39].

Ambler class B “New Delhi-metallo-beta-lactamases” (NDMs) are a transferable type of β-lactamase [40]. NDM-producing *P. aeruginosa* isolates have been isolated in different parts of the world and are considered one of the most common antibiotic resistance mechanisms against carbapenems found in gram-negative bacteria such as *P. aeruginosa* [41]. A study conducted in India confirmed the presence of *bla*_NDM-1_ genes in isolates of *P. aeruginosa* obtained from hospital-acquired infection cases. According to this study [42], the ability of *bla*_NDM-1_ to be transferred from *P. aeruginosa* to other Enterobacteriaceae and vice versa assumed significance in terms of reservoirs of transmissible bacteria or clonal spreads that may be travel-associated [43]. Within a short time, this enzyme has been identified in various regions of the world. In our study, the *bla*_NDM_ gene was identified in seven isolates (21.8%). In other studies, medium to high prevalence was found in analyses also performed on clinical samples. The prevalence rate of 54.55% in carbapenem-resistant PA (CRPA) isolates carrying *bla*_NDM-1_ is the highest among the cross-sectional studies reported from India and Iraq [41]. A study conducted at a tertiary healthcare facility in southwest Nigeria also found a prevalence of 38.4%. These values are in agreement with those of our study and within the values that were verified [44]. A recent study conducted in Korea revealed that *bla*_NDM-1_ was among the most detected metallo-beta-lactamase genes in carbapenem-resistant *P. aeruginosa* isolates. This highlights the significance of *bla*_NDM-1_ in contributing to carbapenem resistance in *P. aeruginosa* strains [45].

Ambler class B “Verona Integron-encoded Metallo-beta-lactamase” (VIM) was first identified in Italy (Verona) in *P. aeruginosa* strains. The *bla*_VIM_ genes are located on mobile gene cassettes inserted in the variable regions of class 1 integrons, a condition that provides a large potential for expression and dissemination in gram-negative pathogens such as *P. aeruginosa* [46,47]. VIM has different type variants, though only the VIM-2 study was performed in this work since it is considered the most frequently acquired MBL gene globally. In our study, we observed the presence of VIM-2 in three *P. aeruginosa* clinical isolates. The spread of this gene can occur rapidly, as this is one of the fastest growing types of MBL genes in the world. The appearance and dissemination of VIM-2-producing *P. aeruginosa* may be a consequence of several factors, such as gene spread among people who may be hospitalized, international tourism, and the intense use of antibiotics, especially in the case of carbapenems. Despite being a gene with low prevalence, studies carried out in hospital environments have detected the presence of this gene in this kind of environment. Isolates obtained from a hospital in Thailand showed a prevalence rate of 12.5% [48].

The virulence genes plcN (12.5%) and pilA (21.9%) showed low prevalence in the clinical isolates. In this study, exotoxin A encoded by toxA was one of the most prevalent virulence factors in *P. aeruginosa* isolates. Studies by Rumbaugh et al. (1999) documented that over 80% of studied isolates from urine produced significant levels of exotoxin A and could thus play an important role associated with urinary tract infections. The toxA gene was observed in 52.4% of the isolates we recovered from urine samples. The algD gene is responsible for alginate production in *P. aeruginosa* and plays a significant role in chronic lung infections [49]. Fourteen *P. aeruginosa* isolates were positive for this gene, with five (33.3%) of them recovered from pneumology samples. Phospholipase C encoded by the plcH gene (hemolytic type) was, in this study, the virulence factor that had the highest prevalence (78.1%) compared to the other genes studied. This gene may also play important roles in the hydrolysis of phospholipids in pulmonary surfactants and is responsible for pro-inflammatory activities, among others. Of the *P. aeruginosa* isolates analyzed, four isolates (16%) from bronchial secretion and expectoration samples were positive for this gene, while three isolates (12%) from blood were also positive. The virulence genes plcN and pilA demonstrated prevalence levels below 50% among our clinical isolates. The plcN gene is a phospholipase C (nonhemolytic type) enzyme, though unlike the plcH gene no pathogenicity effect has been demonstrated [49]. The pilA gene was detected in five *P. aeruginosa* isolates. Our results showed a moderate prevalence of virulence factors (42.5%), and this may depend on several causes, including the nature of the place, the immune status of patients, the degree of contamination, the source of infections, and the type and virulence of the strains [50].

Biofilm-forming bacteria play a significant role in the development of chronic and recurrent infections that pose challenges for treatment. Managing and effectively treating biofilm-associated infections is of utmost importance in healthcare settings [21]. Based on the observations in our results, we do not recommend the CRA method as a suitable method for the detection of biofilm formation [21]. The test was demonstrated to not be effective in the detection of biofilm formation. The lack of positivity in this test may be related to deficient expression of the pel gene responsible for the absent production of the glucose-rich extracellular matrix. The results that were obtained in this study are in agreement with the conclusions made by Lima et al. in 2017. The microplate method was also evaluated in terms of OD_550_, where the microtiter plate method was found to be most effective in the detection of biofilm production [51,52]. When compared to other methods, the microtiter plate method has proven to be the most effective approach for detecting biofilm production. This test offers several advantages, including its simplicity, its utilization of basic laboratory material, and the ability to test multiple samples simultaneously. It allows for both qualitative and quantitative assessment of an isolate’s biofilm-forming capacity. Furthermore, subjective errors are minimized as biofilm formation is measured using a reader, thus reducing the likelihood of observational errors. In this study, all clinical isolates of *P. aeruginosa* were capable of biofilm formation, and the microtiter assay proved to be a reliable and consistent method for screening and detecting biofilm-producing bacteria. This conclusion is supported by studies conducted by other authors who utilized the same methods for biofilm evaluation [52,53,54]. These studies also affirm the accuracy and quantitative capabilities of this technique, making it a recommended approach for assessing biofilm formation in clinical isolates of *P. aeruginosa* [22,55].

ST175 and ST235 are two of the three main high-risk clones. *P. aeruginosa* isolates, resistant to almost all β-lactams, aminoglycosides, and quinolones often ascribed to epidemic clones (more specifically the ST235 or ST111 clones), have been detected in hospitals worldwide. ST235 is certainly considered the most relevant and widespread high-risk clone, being found in many countries across five continents. Indeed, ST235 is the most frequent clone in Portuguese hospitals (REF), whereas in this study it was only observed in two MDR isolates recovered from urine and pus samples. Beyond the strong linkage to multi-resistant phenotypes and horizontally acquired resistance determinants (29), ST235 is characterized by high virulence. The ST235 clone has a wide variety of beta-lactamase variants, including multiple carbapenemases from classes A and B, such as VIM-2, KPC-2, and NDM-1, the most frequent carbapenemases [12]. We can verify in our study that one of the isolates has *bla*_KPC_ and *bla*_NDM_ as its resistance genes. Producers of VIM-2 were analyzed, and one of these producers delivered the international clone ST175, which came from a clinical blood sample. This clone was the most predominant in our study (detected in 11/18 isolates) and was found in isolates from various sources, such as urine, sputum, other liquids, and pus. This turns out to be indicative of the transfer of *P. aeruginosa* between different health care facilities, and this increased risk of cross-transmission and high antimicrobial pressure may have favored clonal spread [13]. ST244 has also been described as a high-risk clone of MDR *P. aeruginosa* worldwide and is associated with multiple different β-lactamases, each with a different geographical spread [15,56]. In our study, it was considered the second most prevalent clone in the isolates and came from three different clinical samples: two blood samples and and bronchial secretion. We can also verify that the samples are considered multidrug resistant. Regarding their virulence, they all have four different virulence factors, that is, they all have similar virulence patterns. Two of the ST244 isolates produced the gene conferring resistance to class A carbapenemases (*bla*_KPC_), and one of the isolates produced the gene conferring resistance to MBLs (*bla*_NDM_). Clone ST2333 was detected in two isolates with different origins (one of orthopedic origin and one from sputum). Both isolates are considered carbapenem-resistant strains and had several virulence factors detected.

Antimicrobial resistance is a growing concern in global health due to its significant impact on various populations. The rise in mortality and morbidity rates across humans and animals is increasingly linked to AR. It is crucial to gain a better understanding of its long-term implications for global health [57].

## 5. Conclusions

*P. aeruginosa* is one of the main gram-negative pathogens and a leading cause of hospital-acquired infections. The indiscriminate and excessive use of antibiotics during infection treatment has led to the development of antibiotic resistance, thus posing a significant public health challenge. *P. aeruginosa* isolates naturally exhibit resistance to many antibiotics. This study focused on molecular analysis of resistance genes and virulence factors in various clinical isolates of *P. aeruginosa*, as well as analysis of their ability to form biofilms. The following conclusions were drawn from this work: Thirty-two clinical isolates from CHTMAD were identified using both phenotypic and genotypic methods, and all were confirmed as *P. aeruginosa*. Multidrug resistance, defined as resistance to at least three different classes of the antibiotics tested, was observed in 93.8% of the isolates. Most isolates demonstrated high resistance to the tested antibiotics, except for amikacin, gentamicin, tobramycin, ceftazidime, and aztreonam, which exhibited a high sensitivity rate. Carbapenem antibiotics showed the highest prevalence of resistance (78.1%), with all 32 *P. aeruginosa* isolates being resistant to imipenem. The studied isolates exhibited a significant capacity for biofilm formation. Additionally, a moderate prevalence of virulence factors (toxA, algD, plcH, plcN, and pilA) was observed, which contributed to varying levels of intrinsic virulence and pathogenicity in *P. aeruginosa*. Furthermore, there is a need for a better understanding of the factors driving the spread of high-risk clones, which can lead to increased mutation rates and enhanced biofilm development. It is crucial to investigate the prevalence of antibiotic-resistant strains and transmission routes to assess the risk of colonization in hospitalized patients.

## Figures and Tables

**Table 1:** Grading of biofilm formation using the microtiter method.

Optical Densities Values OD_550_	Biofilm Formation	Microtiter Method n (%)
>0.240	High	3 (9.0)
0.120–0.240	Moderate	30 (93.7)
<0.120	None	0

**Table 2:** Characteristics of Pseudomonas aeruginosa clinical isolates.

Sample	Time of Collection (Year)	Origin	Resistance		Virulence	MLST ^b^
			Phenotype ^a^	Genotype		
1	2018	Urine	CIP-CN-TOB-FEP-CAZ-DOR-IMI-MRP-PRL-TTC	ND	*toxA- plcH*	NT
2	2018	Urine	FEP-IMI-TTC	ND	*toxA- plcH*	ST235
3	2018	Urine	CIP-FEP-CAZ-DOR-IMI-MRP-ATM-PRL	ND	*toxA- plcH*	NT
4	2018	Healing	FEP-IMI-TTC	ND	*toxA- plcH*	NT
5	2018	Bronchial secretion	IMI-MRP-PRL-TTC	ND	*toxA- plcH*	NT
6	2018	Urine	CIP-FEP-DOR-IMI-MRP-ATM-PRL-TTC	ND	*plcH*	NT
7	2018	Pneumology	FEP-IMI-TTC	*bla*_VIM-2_	*algD*	NT
8	2018	Urine	CIP-FEP-DOR-IMI-MRP-ATM-PRL-TTC	ND	*ND*	NT
9	2018	Urine	CIP-CN-TOB-DOR-IMI-MRP-PRL	ND	*algD*	NT
10	2018	Urine	CIP-FEP-DOR-IMI-MRP-ATM-PRL	ND	*algD*	NT
11	2019	Urine	CIP-CN-TOB-DOR-IMI-MRP-PRL	ND	*ND*	ST175
12	2018	Urine	CIP-FEP-CAZ-DOR-IMI-MRP-ATM-PRL	ND	*plcH*	ST175
13	2018	Blood	IMI-MRP-TTC	*bla*_VIM-2_	*plcH*	ST175
14	2018	Urine	FEP-IMI-TTC	ND	*toxA*	ST175
15	2018	Urine	AK-FEP-IMI-TTC	ND	*toxA- plcH algD*	ST175
16	2018	Urine	CIP-CN-TOB-FEP-CAZ-IMI-ATM-PRL	*bla*_KPC_-*bla*_NDM_	*toxA- plcH*	ST235
17	2018	Orthopaedics	FEP-IMI-MRP-TTC	*bla*_NDM_ -*bla*_VIM-2_	*toxA- algD- plcH- pilA*	ST2333
18	2018	Expectoration	CIP-CN-TOB-FEP-DOR-IMI-PRL	*bla*_KPC_	*toxA- algD*	ST2333
19	2018	Urine	FEP-CAZ-DOR-IMI-MRP-ATM-PRL-TTC	ND	*toxA- plcH*	NT
20	2018	Expectoration	CIP-FEP- CAZ-DOR-IMI-MRP-ATM-PRL	ND	*toxA- plcH- algD- pilA*	ST175
21	2019	Blood	TOB-FEP-DOR-IMI-MRP-TTC	*bla*_KPC_	*toxA- algD- plcH*	NT
22	2019	Urine	CIP-TOB-FEP-CAZ-DOR-IMI-MRP-PRL-TTC	*bla*_NDM_	*toxA- algD- plcH- plcN*	ST244
23	2019	Urine	CIP-CN-TOB-FEP-IMI	*bla*_NDM_	*toxA- algD- plcH*	ST175
24	2019	Urine	CIP-CN-TOB-IMI-PRL	*ND*	*toxA- plcH*	NT
25	2019	Bronchial secretion	CIP-FEP-CAZ-DOR-IMI-MRP-ATM-PRL	*bla*_KPC_	*algD- plcH- plcN- pilA*	ST244
26	2019	Blood	CIP-CN-TOB-FEP-DOR-IMI-MRP-PRL	*bla*_NDM_	*toxA- algD- plcH- pilA*	ST244
27	2019	Pus	CIP-CN-TOB-FEP-DOR-IMI-MRP	*bla*_NDM_	*toxA- algD- plcH- plcN*	NT
28	2019	Drainage liquid	FEP-CAZ-DOR-IMI-ATM-PRL-TTC	*bla*_KPC_	*toxA- plcH- pilA*	NT
29	2019	Expectoration	FEP-CAZ-DOR-IMI-MRP-ATM-PRL	*bla*_NDM_	*algD- plcH*	ST175
30	2020	Other liquid	CIP-FEP-DOR-IMI-MRP-PRL	*bla*_KPC_	*toxA- plcH- pilA*	ST175
31	2020	Urine	CIP-FEP-DOR-IMI-MRP-ATM-PRL	*ND*	*toxA- plcH- plcN- pilA*	ST235
32	2020	Pus	CIP-CN-TOB-FEP-DOR-IMI-MRP	*bla*_KPC_	*plcH*	ST175

Legend: ^a^ PRL: piperacillin; CAZ: ceftazidime; DOR: doripenem; IMI: imipenem; MRP: meropenem; CIP: ciprofloxacin; CN: gentamicin; TOB: tobramycin; FEP: cefepime; ATM: aztreonam; TTC ticarcillin-clavulanic acid. ^b^ MLST—multilocus sequence typing; NT: not tested; ND: not detected.

## Data Availability

All data related to this research are contained within this paper.
